# Supplementary material for: Positive Association of Serum Alkaline Phosphatase Level with Severe Knee Osteoarthritis: A Nationwide Population-Based Study
Source: Diagnostics (Basel). 2020 Nov 27;10(12):1016. doi: 10.3390/diagnostics10121016 (PMC7760969; doi:10.3390/diagnostics10121016)
Supplement: Supplementary file 1 [file diagnostics-10-01016-s001.zip › Figure S1. percentage of participants with weight status according to the severity of osteoarthritis.pptx]

## Slide 1
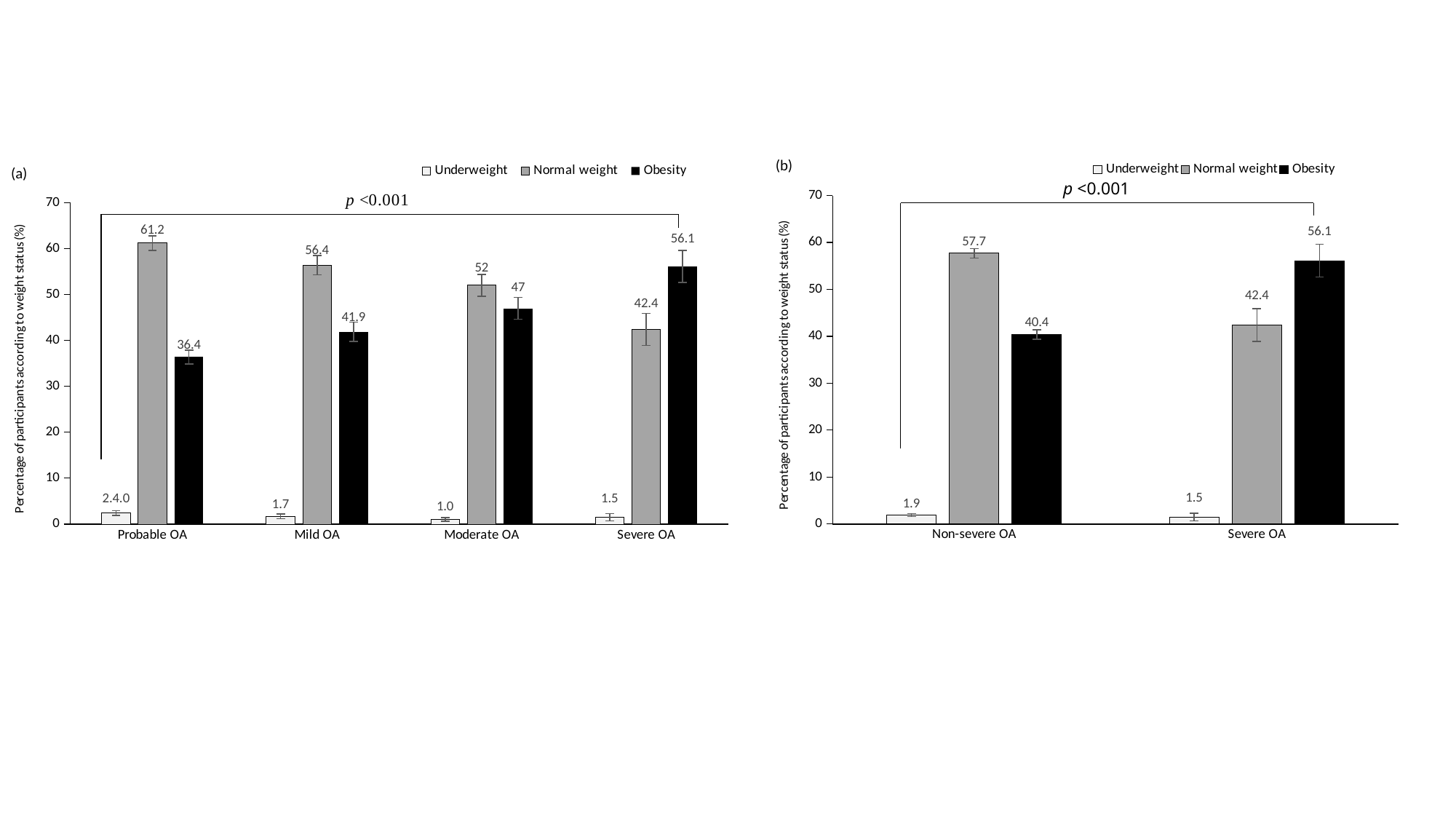

### Chart
| Category | Underweight | Normal weight | Obesity |
|---|---|---|---|
| Non-severe OA | 1.9 | 57.7 | 40.4 |
| Severe OA | 1.5 | 42.4 | 56.1 |(b)
### Chart
| Category | Underweight | Normal weight | Obesity |
|---|---|---|---|
| Probable OA | 2.4 | 61.2 | 36.4 |
| Mild OA | 1.7 | 56.4 | 41.9 |
| Moderate OA | 1.0 | 52.0 | 47.0 |
| Severe OA | 1.5 | 42.4 | 56.1 |(a)
p <0.001
