# Supplementary material for: Positive Association of Serum Alkaline Phosphatase Level with Severe Knee Osteoarthritis: A Nationwide Population-Based Study
Source: Diagnostics (Basel). 2020 Nov 27;10(12):1016. doi: 10.3390/diagnostics10121016 (PMC7760969; doi:10.3390/diagnostics10121016)
Supplement: Supplementary file 1 [file diagnostics-10-01016-s001.zip › Figure S2. mean value of BMI according to the severity of osteoarthritis.pptx]

## Slide 1
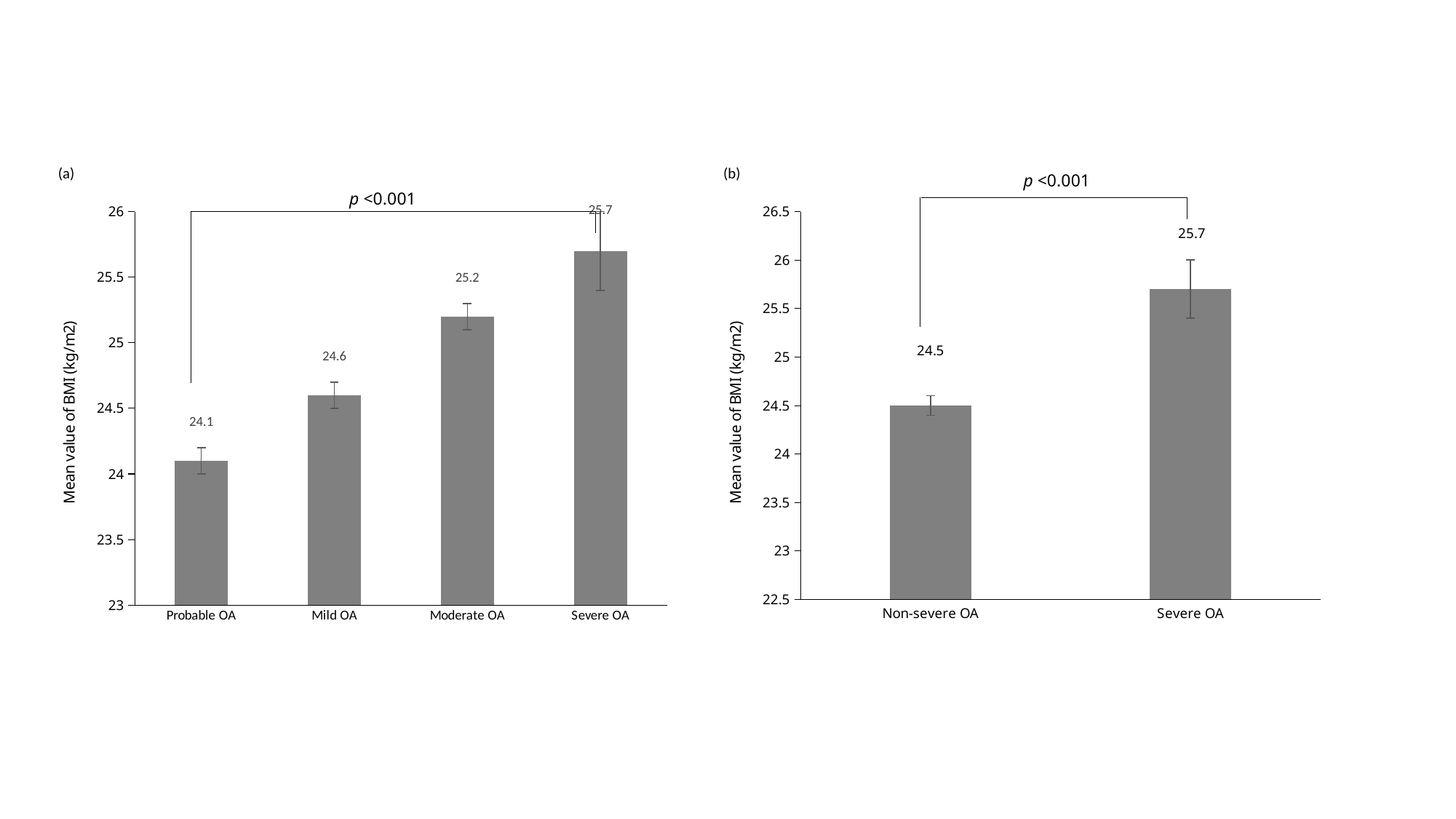

(a)
(b)
p <0.001
p <0.001
### Chart
| Category | mean value of BMI |
|---|---|
| Non-severe OA | 24.5 |
| Severe OA | 25.7 |
### Chart
| Category | mean value of BMI |
|---|---|
| Probable OA | 24.1 |
| Mild OA | 24.6 |
| Moderate OA | 25.2 |
| Severe OA | 25.7 |
